# Universal health coverage in Rwanda: dream or reality

**DOI:** 10.11604/pamj.2014.17.232.3471

**Published:** 2014-03-27

**Authors:** Médard Nyandekwe, Manassé Nzayirambaho, Jean Baptiste Kakoma

**Affiliations:** 1 School of Public Health, National University of Rwanda, Butare, Rwanda

**Keywords:** Universal Health Coverage, Community-Based Health Insurance, Rwanda

## Abstract

**Introduction:**

Universal Health Coverage (UHC) has been a global concern for a long time and even more nowadays. While a number of publications are almost unanimous that Rwanda is not far from UHC, very few have focused on its financial sustainability and on its extreme external financial dependency. The objectives of this study are: (i) To assess Rwanda UHC based mainly on Community-Based Health Insurance (CBHI) from 2000 to 2012; (ii) to inform policy makers about observed gaps for a better way forward.

**Methods:**

A retrospective (2000-2012) SWOT analysis was applied to six metrics as key indicators of UHC achievement related to WHO definition, i.e. (i) health insurance and access to care, (ii) equity, (iii) package of services, (iv) rights-based approach, (v) quality of health care, (vi) financial-risk protection, and (vii) CBHI self-financing capacity (SFC) was added by the authors.

**Results:**

The first metric with 96,15% of overall health insurance coverage and 1.07 visit per capita per year versus 1 visit recommended by WHO, the second with 24,8% indigent people subsidized versus 24,1% living in extreme poverty, the third, the fourth, and the fifth metrics excellently performing, the sixth with 10.80% versus ≤40% as limit acceptable of catastrophic health spending level and lastly the CBHI SFC i.e. proper cost recovery estimated at 82.55% in 2011/2012, Rwanda UHC achievements are objectively convincing.

**Conclusion:**

Rwanda UHC is not a dream but a reality if we consider all convincing results issued of the seven metrics.

## Introduction

Universal Health Coverage (UHC) has been a global concern for a long time. It is firmly based on the World Health Organization (WHO) constitution of 1948 declaring health a fundamental human right and the Health for All agenda set by the 1978 Alma-Ata Declaration that Rwanda ratified. WHO defines Universal Health Coverage as ensuring that all people have access to needed promotive, preventive, curative and rehabilitative health services, of sufficient quality to be effective, while also ensuring that the use of these services does not expose the user to financial hardship [1].

This WHO definition of UHC embodies three related dimensions: Equity in access to health services, i.e. those who need the services should get them, not only those who can pay for them; Quality of health services, which is good enough to improve the health of those receiving services; Financial-risk protection i.e. universal coverage must bring the hope of better health and protection from poverty for hundreds of millions of people, especially those in the most vulnerable situations.

UHC in health care, as determined solely by financing mechanisms and access to healthcare services has been achieved in 27 member states of the Organization for Economic Cooperation and Development (OECD) [2]. Encouraging progress has also been made in some low and middle-income countries, including Thailand, Sri Lanka, Rwanda, Cuba, Colombia and Chile [3].

According to 2010 World Report, the concept is taking off in countries as varied as South Africa, India, Rwanda, Indonesia, and the United States, with governments around the world engaging in serious political and technical discussions on how to expand the Universal Health Coverage[4]. Except for the United States, 25 wealthiest nations now have some form of UHC. Others are not far behind, including Brazil and Thailand. Even nations at lower income levels, such as Philippines, Vietnam, Rwanda and Ghana are working towards it. South Africa, China, and Colombia are on the move too [5].

For Sub-Saharan Africa countries, including Rwanda, UHC remains an important challenge, with millions of households struggling with high percentage of Out-Of-Pocket (OOP) in total household expenditure for health services [6].

Since September 2012, Rwanda was recognized as one of the nine countries in Africa and Asia making significant progress to make universal healthcare systems possible. The five countries on intermediate - stage reform are Ghana, Indonesia, Philippines, Rwanda, Vietnam and the four on early-stage reform are India, Kenya, Mali and Nigeria [7].

**Figure 1:**
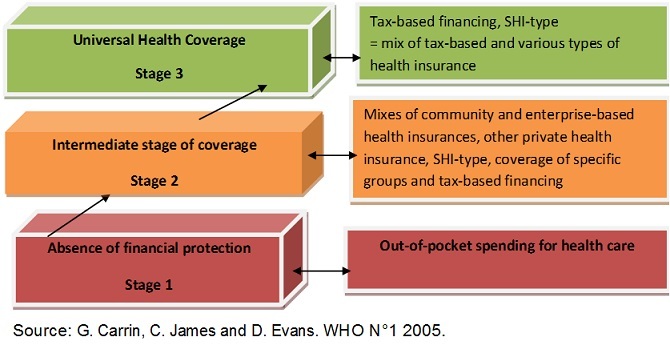
The transition to universal health coverage

According to Hsiao 2003 cited by Jesse B. Bump 2010 [8], until September 2012 the UHC built on CBHI had been observed nowhere in the world; the model of Rwanda UHC would be therefore the first of the kind. According to G. Carrin et al [9], the transition to Universal Health Coverage starts from stage 1 where there is no financial protection to stage 3 where universal coverage is effective (Figure 1).

Considering different stages of transition to UHC as proposed by G. Carrin et al., the results of the study will be used to range Rwanda UHC. Five basic factors highlight the Rwanda commitment to move towards UHC: The long-term strategy Vision 2020 with a strategic social protection through universal access to health care (2000) [10]; The “Rwanda's Politique Nationale de Développement des Mutuelles”(2004) [11]; The Law Nº 62/2007 of December 30th promulgated in March 2008 [12] which states that all Rwandan residents must be affiliated to a health insurance scheme that provides quality health care; The Rwanda Community Based Health Insurance Policy 2010 [13]; The Rwanda National Health Insurance Policy 2010 [14].

In line with this latter law, CBHI became mandatory to informal sector population and joined other Rwanda's Social Health Insurances (La Rwandaise d'Assurance Maladie or RAMA and Rwanda Military Medical Insurance or MMI). According to Mid Term Review (MTR) of the Rwanda Second Health Sector Strategic Plan (HSSPII) [15], Rwanda was about to reach Universal Health Coverage at least in 2011. While published articles are almost unanimous that Rwanda is not far from UHC [2–7, 15–18] very few have focused on its financial sustainability [19–23].

This article makes a critical scrutiny of Rwanda UHC according to Stuckler's metrics [23] including also CBHI financial sustainability through its self financing capacity, and examines whether Rwanda UHC is a reality or rather a dream, in the context where its main component (CBHI) is covering 90.75% of population i.e. approaching the 91% reached from 2010 [24].

The objectives of this study are: To make a critical scrutiny of Rwanda UHC based mainly on CBHI from 2000 to 2012 by highlighting its progress, strengths, weaknesses, opportunities and threats; To inform policy makers about observed gaps for a better way forward.

## Methods

This is a national retrospective review using CBHI data from 2000 to 2011/2012 fiscal years for seven metrics below. The Rwanda CBHI was assessed through a rigorous SWOT analysis according to six metrics developed by Stuckler D et al. [23] with reference to WHO definition of UHC. Besides, the SFC as a seventh metric has been added on for this study by the authors in order to measure Rwandan CBHI financial sustainability. Concerning the SFC SWOT analysis, the study has only considered one Fiscal Year (FY) period 2011/12 for assessing the CBHI financial sustainability from the implementation of the new CBHI policy on 1st July, 2011. The seven metrics have been used to serve as evidence based assessment tool on how Rwanda CBHI complies scientifically and objectively with UHC prerequisites:

1. Access to care and Health Insurance: Basically, the provision of UHC assumes that everyone can: a) get health insurance b) access health services, and c) have access to care with financial risk protection. Assessment of access to health care is usually based on HCs’ utilization and assisted deliveries.
2. Equity in access to health services: “The Equity in access to health services dimension means that those who need the health care should get them regardless of ability to pay”. Equity is also to be defined as equal access to benefit package irrespective of one's socio-economic status and risk equalization meaning the financial risk of illness is equally shared among all citizens.
3. Rights-based approach: every individual has the right to essential primary health care. The UHC is linked firmly to the right to essential health care that is to access prevention, essential drugs and primary health care.
4. Package of Services: coverage and affordability including first line referral hospital to ensure appropriate back up for first line care, rather than only being understood as first line care is recommended.
5. Quality of health care: the quality of health services should be good enough to improve the health of those receiving services.
6. Social and economic/financial- risk protection: effective access to affordable health care of adequate quality and financial protection in case of sickness. This metric was assessed through the calculation of households Out-Of-Pocket spending in healthcare.
7. CBHI self-financing capacity (SFC): according to Business Dictionary, SFC is the surplus cash generated at the end of a fiscal year. The more the SFC is higher, the more growth opportunities are important. Conversely, a negative SFC reflects a highly critical situation. The authors used it as a complementary tool to measure the performance of CBHI's financial sustainability.

This study will serve as baseline assessment in order to provide evidence based indicators in monitoring and evaluation of Rwanda UHC progress.

## Results

### First metric: insurance and access to care metric

**Figure 2:**
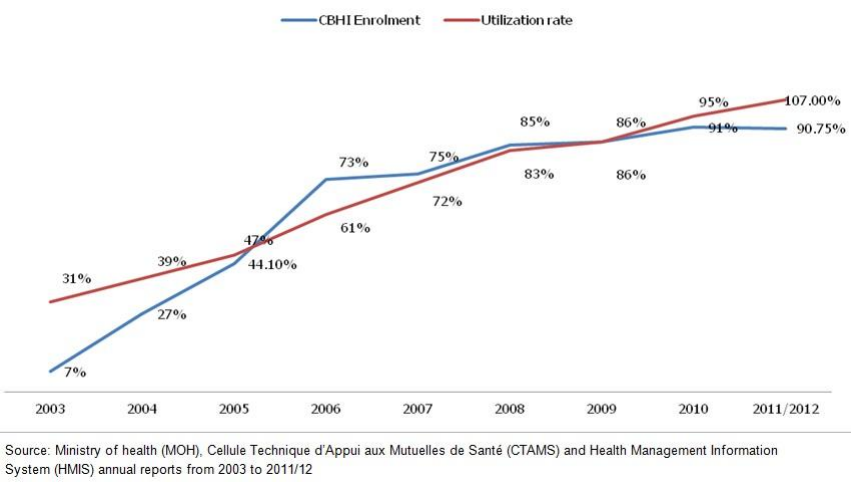
Trends in Community-based health insurance coverage and health centers utilization

The two first sub-dimensions of the first metric i.e. “get health insurance ” and “access health service” for Rwanda are illustrated by the Figure 2.

### Insurance sub-metric

**Figure 3:**
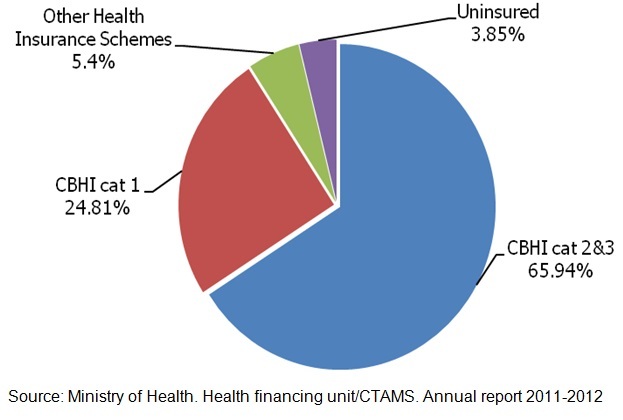
Rwanda health insurance coverage for year 2011-2012

**Strengths:** increase in CBHI coverage since 2003 (7%) to 2010 (91%) with a light inflexion to 90.75 % in 2011/2012 [17], so that the overall health insurance coverage at national level is 96.15% including other health insurance schemes (5,4%) as illustrated by Figure 3; The Law n° 62 / 2007 of 30/12/2007 governing CBHI makes provisions for all Rwandan to access health Insurance; *Ibimina* (tontines) as a mechanism of premiums collection.

**Opportunities**: Strong government leadership, political commitment, good governance and stewardship; Synergy between reforms in health sector; Culture of solidarity and mutual assistance and/or aid; Inclusion of CBHI enrolment in districts’ performance contracts between local governments and the President of the Republic of Rwanda (Imihigo).

**Threat faced by CBHI's system:** economic constraints: according to “Third Integrated Household Living Conditions Survey (EICV3 2010/1011)” [25]. Almost half (44.9%) of Rwanda population lives below poverty line, and 24.1 % of population live in extreme poverty.

### Utilization of health services sub-metric

**Strengths:** progressive increase in access health care from 31% (in 2003) to 107 % (in 2012) which is close to the WHO recommendation (1 visit per capita per year); Deliveries assisted by a skilled birth attendant increasing from 52% [15, 26] to 66.3% in year 2009[27, 28]; Patient roaming system consisting in allowing registered CBHI members with a validated card to access health care in any public and Faith Based Organizations across the country [24].

**Weakness:** Financial barriers for the poor category (UBUDEHE category 3 equivalent to the lower category of CBHI category 2) that finds it difficult to pay premium.

### Second metric: equity and financial-risk equalization

**Strengths**: improvement of equity in the community health insurance system: 24.8% of subsidized vulnerable in 2011/2012 versus 24.1% living in extreme poverty. Premiums contribution is progressive in the new policy determined by socio economic category one belongs to from the implementation of New CBHI policy on 1st July, 2011: During the implementation of the old CBHI policy i.e. from 2005 to June 2011, the premium was a flat rate of Rwandan Francs (RWF) 1,000 (US$1.67) per capita with a subsidy of the same amount from the government and development partners.

The new stratified premium system which took effect from July 1, 2011was based on ‟UBUDEHE‚ criteria as presented in Table 1 [13, 14]. The ‟UBUDEHE‚ Approach is community based system that assesses the financial situations of citizens living in villages throughout Rwanda. The community evaluates each household's or citizen's financial asset situation and places it in one of six socio-economic categories. It is a part of the Vision 2020 Umurenge Programme (VUP) that aims to improve the lives of citizens and promote community development from the bottom up instead of top down.

**Table 1:** New stratified premium system used by new community-based health insurance policy

CHBI category	Population coverage	Premium
Group 1: Very Poor category (Ubudehe category 1 and 2)	24.8%	RWF 2,000 (US$3.34)
Group 2: Poor category (Ubudehe category 3 and 4)	68.8%	RWF 3,000 (US$5)
Group 3: Rich category (Ubudehe category 5 and 6)	2.17%	RWF 7,000 (US$11.69)

Source: Rwanda CBHI Policy, 2010

Improvement in risk equalization between the socio-economic categories. It is worth mentioning that the CBHI utilizes ‟UBUDEHE‚ Approach as defined by Rwanda Ministry of Local Administration (MINALOC) and whose categories are presented in Table 1.

Improved risk equalization between CBHI levels and re-distribution of sections’ financial reserves year after year.

**Weakness:** difficulties faced by the population in lower category of poor persons to pay the annual premium in one go.

**Threat:** weak financial-risk sharing between CBHI members and affiliated of other health insurance companies [29].

### Third metric: rights-based approach of UHC

**Strength:** CBHI in Rwanda is compulsory and all CBHI members have an equal right to insurance benefits offered through public and faith-based health facilities (HF) i.e. benefit from almost the whole domestic quality health care provision.

**Weakness**: The right to essential health care is a new concept in Rwanda; patients do not know their right with regards to healthcare; Generally, patients do not claim when some issues are not solved by health providers.

### Fourth metric: package of services

**Strength**: Comprehensive health care benefits package from Health Center (HC) to referral hospitals in public and faith-based health facilities.

**Weaknesses**: No access to certain treatments; No access to private health providers.

**Opportunity:** Existence of national advocacy of additional essential health services (circumcision, prostheses, eyeglasses, etc) but not yet included in the current package.

### Fifth metric: quality of health care

**Strengths**: Synergy between CBHI, PBF and Quality Assurance (QA) in improvement of quality health care [16]; Commitment of the Ministry of Health to roll out a policy on health strengthening geared to improve the quality of health care by accreditation of HF;

**Weaknesses**: Despite this policy above, poor quality of health services is observed by recent evaluations [15]; Some CBHI members declare at various media that they are not satisfied by poor courtesy, the weak customer care and the regular stock-outs of drugs in some contracted health facilities; Sometimes incapacity or delay in reimbursement of health care bills have been observed [15]; Occurrence of incapacity at hospital pharmacies level to serve all drug prescriptions due to stock-outs [23]. Such situations affect quality of care for CBHI members particularly the costumer care aspect.

### Sixth metric: social and economic risk protection/financial risk- protection

**Strengths**: The 10.80 % observed level of catastrophic health spending that Rwandese were faced with in year 2010 (EICV3) is to be considered as OOPs payments at an acceptable level according to the related indicator, i.e. ≤ 40% [17]; Improved catastrophic health spending illustrated by Total Households Out of pocket Health Expenditure (HHOOPHE) as a percentage of Total Health Expenditure that dropped from 23% in fiscal year 2006 to 15% in fiscal year 2010 [15].

**Weakness**: Low level of financial-risk protection. HHOOPHE estimated at 15% of Total Health Expenditure (THE) are almost equal to government contribution to the health sector (i.e.16% of THE in 2010/2011)[30]: this portrays a big financial burden that population and especially CBHI members are still bearing in term of OOPs health spending which consequently represents a low financial-risk protection in case of illness. Progressive increase of tariffs practiced on health care and co-payment in case of illness, expose CBHI members to financial-risk especially at district and referral hospitals where bills are relatively important (10% of the billable cost of services).

### Seventh metric: self-financing capacity (SFC)

**Figure 4:**
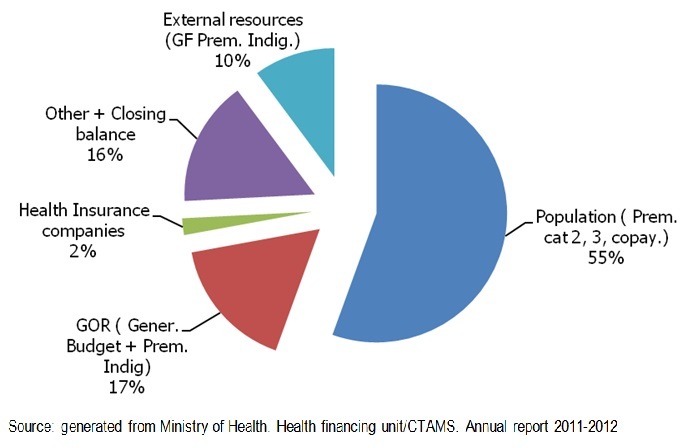
Community-based health insurance sources of financing

The CBHI Self financing capacity is assessed through the comparison between the CBHI income estimated at RWF 25,337,575,163 equivalent to US$ 39 589 961 (1US$=RWF 640 in July 2013) and expenditure estimated at RWF 23,312,007,180 equivalent to US$ 36 425 011 for the fiscal year 2011/2012. The CBHI sources of financing for the Fiscal Year 2011/2012 are illustrated by Figure 4.

**Strengths**: Increased mobilization of domestic resources to ensure CBHI financial independency; Decreased misappropriation of funds at section level.

**As positive consequences:** The very good Self-Financing estimated at 82.55% and the very good total recovery cost estimated at 108.69% auguring an incoming financial sustainability of CBHI Scheme.

**Weaknesses**: Overuse, over-prescription and over-charging of health services; Increase in pharmaceutical and medical surgical consumables prices due to inflation rate will continuously influence a negative SFC; CBHI poor financial management especially weak cost control.

**Opportunity**: Existence of other legal resources not yet allocated to CBHI (1% TVA, 5% cross-subsidy contribution from other insurance companies, etc.).

## Discussion

As the results of the first, third, fourth and fifth metrics are quite clear and consistent with the reality; the discussion will focus on relevant aspects of the last three metrics, namely equity and risk equalization, social and economic risk protection or financial-risk protection and SFC.

### Second metric: equity and financial-risk equalization

The equity with 24.8 % of population whilst ± 24.1 % of population living in extreme poverty, with the progressive premium contribution introduced by the new CBHI policy, the fairness and equity are demonstrated. Despite this very good performance, people in lower stratum of CBHI category 2 i.e. in “UBUDEHE” cat 3 are facing difficulties to pay the annual premium in one go. Consequently they cannot access health care during the discontinuance period as long as they haven't paid the total individual premium for the whole nuclear family. As for the financial-risk equalization aspect, disparities do exist between the formal sector health insurances and CBHI members; there is a lack of risk sharing mechanisms between CBHI Scheme and other health insurance companies.

### Sixth metric: social and economic risk protection or financial-risk protection

The 10.80% of catastrophic health spending faced by Rwandese is to be considered as OOP payments at an acceptable level because the level becomes unacceptable from ≥40% [17]. According to recent publications presented as thorough researches [17, 18], one could think that Rwanda is moving backward in financial-risk protection from the scale-up of mutuelles. In effect, Fernandes Antunes et al. [17] have estimated this indicator at 7% of catastrophic health spending in year 2009, distributed differently according to wealth categories: 15.5% in very poor category (Q1) and 3.8% in wealthy category (Q5).

Chunling Lu et al. [18] have determined the percentage of total Rwanda household with catastrophic health spending at 11.9% in Year 2000. For the Year 2006 and by logistic regression method, the same authors have determined this indicator at 10.5%, 7.7% and at only 5.1% respectively in uninsured people, in total population and insured people including CBHI members.

Since its inception, CBHI has offered numerous advantages including behaviour change in health investment and especially in using health services when needed as direct impact followed by better health status. Other observed direct impacts are the following: CBHI influences HH's decision making and behavior to seek and access medical care. This is proved by the increased health services utilization among insured compared to non-insured [31, 32]; The CBHI patient members’ roaming system shall positively influence CBHI members’ behaviour, e.g. to consult any contracted health provider and anywhere in case of illness. The HHOOPHE level shall be higher than previously because the co-payment contribution will be paid for each visit. Meaning that an increase is awaited but it shall not be considered as moving backward in catastrophic health spending alleviation.

The OOP expenditure (i.e. 15% of Total Health Expenditure/THE) is almost equal to government contribution to the health sector (i.e. 16% of THE in 2010/2011); this is an indicator of the financial-risk faced by households in case of illness.

The explanation of recent and incoming increase of catastrophic health spending level is probably the positive impact of CBHI in health care seeking.

## Seventh metric: self-financing capacity

Analyzing Rwanda CBHI SFC, the authors have observed a good picture of Rwanda CBHI financial statement for FY 2011/2012. This is illustrated by the very good SFC in the Rwanda economic context i.e. proper cost recovery estimated at 82.55%, the internal cost recovery and the overall cost recovery estimated at 97.72% and 108.69% respectively. Thus, one should recognize that this metric shows a very good score through these convincing financial indicators. Therefore, the CBHI Scheme has proved to be financially sustainable. Despite this very good performance, the study revealed a very high overhead (administrative costs) estimated at 18.71% of total expenditure. This should be qualified as a weak cost control. Ranu S Dhillona & al. [20], have observed the same problem of administrative cost: 'Administering the CBHI program itself, recent data show that it requires a lot of overhead cost and diverts government funds’.

In other countries, administrative expenses engaged by different social and community based health insurance schemes were comprised between 5% and 12% in year 2004. For instance the pilot prepayment schemes in Rwanda spent 7%-10% for overhead; while CBHI system of Western Africa assisted by PHRplus spent 5%-10%. In OECD countries, social health insurances spent between 5% -7% and the USA health insurance sector spent in average 12% of total revenues, what is qualified as too high by authors [21].

According to David Stuckler et al. [23], the authors who have developed the six metrics applied in this study, the Rwanda CBHI can't be replicable in other places or in other countries of same socio-economic status because “it reflects an ‘out-of-the-box’ model evolving in resource-poor settings with decentralized government decision-making and small patchworks of disintegrated clinics depending on foreign assistance”.

The same authors pursued: “Immediately several questions are raised about Rwanda's experience: Is this UHC? Does it qualify as a Universal Health Care? Should its model be encouraged to countries in similar economic positions? If so, given its relatively privileged situation as a recipient of development assistance, how replicable is this model in seeking to ‘graduate’ from donor support to independent provision of UHC?”

Another author raised a question about the new progressive contribution model qualifying CBHI as a social health insurance for the poor and sharing pooling for the poor: “Rwanda is now introducing higher premiums to gain more finances through the program; but charging the poor to cover more of the poor, and pricing them out of reach, it is unlikely to produce substantial new funding or health improvements” [22].

According to this study's findings, which show a very good financial picture of CBHI on which Rwanda UHC is built, the authors disagree with those who raise the question of the non financial sustainability and the non reproducibility of Rwanda CBHI because the later is financially viable now if we consider its current SFC estimated at 82.55% in FY 2011/2012 where the new CBHI policy with progressive premiums based on socio economic categories is implemented.

With regards to this self financing capacity (SFC), and the non specified revenues generated or allocated to CBHI in the same fiscal year, the revised score should jump to 97.72%.

Other revenues, for instance the 1% of VAT (+/- 6 billion for the FY 2011/2012) and the only 1% (RWF 530,384,098) instead of 5% coming from other health insurance companies are among others legal proper revenues not yet allocated to CBHI. The new CBHI law has determined also other plausible sources of financing (specific tax on alcohol, on cigarettes and extending cross-subsidy contribution to other private insurance companies).

These measures will certainly provide a lot of additional income ensuring CBHI self financing capacity (financial independency) and therefore its financial sustainability.

## Conclusion

According to WHO definition and to Stuckler's metrics in line with WHO definition, the Rwanda Universal Health Coverage is not a dream but is a reality if one considers the findings of the study. According also to G. Carrin, C. James and D. Evans et al. UHC's transition figure, current Rwanda UHC is to be ranged between the intermediate stage II of coverage and the stage III of effective UHC because some gaps have been observed at the second, sixth and seventh metrics. In fact, as regards the six Stuckler's metrics, they are either excellently (1^st^, 3^rd^, 4^th^, 5^th^) or very well performing (2^nd^ and 6^th^). With regards to the seventh metric, the SFC with a performance of 82.55% as proper total cost recovery is a very good indicator of financial sustainability because some budget lines non specified have been gathered in the only one “other revenues” whilst they seem to have been generated by the efficient FY 2011/12 CBHI implementation. Thanks to these other revenues and to GOR's legal yearly contribution, the internal total cost recovery has attained 97.72% illustrating the almost CBHI financial independency. Despite the non exaggerated weak cost control revealed by the study, CBHI financial sustainability is proven. In order to overcome some gaps highlighted by the study, i.e. financial risk-equalization, financial-risk protection, CBHI poor financial management, and increase the CBHI self-financing level, the authors suggest that the Government of Rwanda hastens a study on how inequalities in access to quality health care can be strongly reduced and current mechanisms of financial-risk protection can be more improved; Gradually improves CBHI SFC through an increase of domestic financial resources; Enhances better costs control for all mobilized resources and set up system regulation in the health insurance system and especially in CBHI scheme; Sets up mechanisms of merging Government owned social health insurances from formal sector (RAMA and MMI) that have a proven management capacity with CBHI for a better costs control , a better common and national financial -risk sharing and a better one financial sustainable national pool as stated in the Rwanda National Health Insurance Policy 2010.
